# Dispositional Self-Construal Modulates Neural Representation of Self: An ERP Study

**DOI:** 10.3389/fpsyg.2020.00895

**Published:** 2020-05-26

**Authors:** Jie Chen, Panpan Yuan, Yaohan Cai, Cuihong Liu, Wenjie Li

**Affiliations:** ^1^School of Educational Science, Hunan Normal University, Changsha, China; ^2^Cognition and Human Behavior Key Laboratory of Hunan Province, Changsha, China

**Keywords:** dispositional self-construal, independent, interdependent, self-name, P2

## Abstract

This study aimed to investigate the influence of dispositional self-construal on self-related processing. Event-related potentials (ERPs) were recorded for a participant’s own and a famous person’s name in a three-stimulus oddball task. The results showed greater P2 and P3 amplitudes induced by one’s own than by a famous person’s name in both independent and interdependent self-construal groups. However, no N2 amplitude differences were found between the partcipant’s own name and a famous person’s name in either group. Moreover, the strength of the P2 effect (own vs. famous person’s name) was stronger in the independent than in the interdependent self-construal group, whereas the P3 effect was similar between these two groups. Thus, these findings might reflect fast modulation of self-related processing by dispositional self-construal.

## Introduction

Self-construal refers to how one defines oneself in relation to others (Markus and Kitayama, 1991). Recent studies have demonstrated a widespread influence of self-construal on multiple cognitive and emotional processes, including attention (Lin and Han, 2009; Liddell et al., 2015; Liu et al., 2015), empathy (Jiang et al., 2014; Wang et al., 2014), interpersonal relationships (Holland et al., 2010; Wang et al., 2015), and so on. Moreover, the association between self-construal and self-cognition has been an important topic for researchers (Sui et al., 2013; Polman and Vohs, 2016; Steinmetz and Mussweiler, 2017).

Previous studies found that self-construal could modulate self-related processing in several aspects, such as self face recognition and self-evaluation. For example, an event-related potential (ERP) study showed that priming self-construal can modulate neural responses to self-cognition in a face-orientation judgment task. British subjects primed with interdependence showed reduced attention toward their own faces, as indexed by smaller N2 amplitudes, and Chinese subjects primed with independence showed reduced attention toward friends’ faces, also indexed by smaller N2 amplitudes (Sui et al., 2013). In addition, self-evaluation under social threat could be affected by self-construal. Specifically, subjects primed with independence demonstrated a greater above-average effect (emphasizing own desirability) under social threat but not those primed with interdependence (Zhang et al., 2017). Moreover, individuals primed with independence had greater feedback-related negativity (FRN), a negative deflection sensitive to outcome evaluation, when responding to outcome feedback about themselves than did their mothers during a gambling task (Zhu et al., 2018) and no such effect was observed in individuals primed with interdependence, indicating that priming independent self-construal could enhance sensitivity to one’s rewards. Additional to these ERP studies, functional magnetic resonance imaging (fMRI) studies have provided further evidence that the medial prefrontal cortex (MPFC) regions showed greater activation after priming independent self-construal (Chiao et al., 2010). Generally, these studies suggested that neural responses to self-related processing could be modulated by priming temporary self-construal.

Although previous studies have provided a good understanding of the effects of self-construal on self-related processing, they mainly focus on the effects of self-construal priming. Although priming techniques may likely activate the associated self-construal and make it temporarily accessible (Cross et al., 2011; Zhu et al., 2017, 2018), temporary situational self-construal may not be equal to dispositional self-construal. The dispositional self-construal is a stable trait, the formation of which is influenced by long-term cultural experiences (Sui et al., 2013). An fMRI study showed that self-relevant processing within MPFC could be modulated by dispositional self-construal measured by the self-construal scale (SCS) (Chiao et al., 2009). In addition, it has been suggested that the commonly used priming tasks may induce specific ways of thinking or feeling that are associated with particular behaviors but not with self-construal (Cross et al., 2011). Thus, this study directly measured the dispositional self-construal by the SCS (Singelis, 1994) and aimed to find different temporal features underlying self-representation in individuals with independent and interdependent self-construals. Although East Asians are characterized by interdependent self-construal and Westerners are characterized by independent self-construal (Markus and Kitayama, 1991), it was suggested that both independent and interdependent self-construals existed in each culture (Oyserman et al., 2002). Thus, this study was performed in order to explore whether dispositional self-construal could modulate neural activities to self-related processing in the eastern culture and also to explore differences in the temporal features of self-related processing between individuals with independent and interdependent self-construals.

It is considered that a participant’s own name is an inherent part of the self-concept and plays an important role in everyday life (Kang, 1972; Watson, 1986). Moreover, self-name has often been considered an ideal experimental stimulus to investigate the neural processing of self-related stimuli (Tacikowski and Nowicka, 2010; Chen et al., 2011, 2013; Tacikowski et al., 2014). Thus, participants’ own names were selected as the self-relevant stimulus and a famous person’s name as the familiar stimulus. In order to make the participant’s own name appear unexpectedly, the three-stimulus oddball paradigm was adopted, and subjects were asked to respond only for the target stimulus (small circle) in the stream of standard stimuli (big circles) and distractors (the participant’s name and that of a famous person) (Chen et al., 2015). Moreover, we decided to use the ERP technique, which has high temporal resolution, to investigate the temporal features underlying the influences of dispositional self-construal on self-related processing, as this cannot be unraveled with fMRI due to its low temporal resolution.

Prior ERP studies showed that P2, N2, or P3 amplitudes could be modulated by self-related stimuli. For example, larger P2, P3, or smaller N2 amplitudes were evoked by own than by others’ names (Tacikowski and Nowicka, 2010; Chen et al., 2011). It is well known that individuals with independent and interdependent self-construals emphasize their uniqueness and interpersonal relationships, respectively (Markus and Kitayama, 1991; Cross et al., 2011). Considerable research has indicated that, after independent self-construal priming, individuals responded more strongly to self-related stimuli than did individuals with interdependent self-construal priming (Sui and Han, 2007; Chiao et al., 2010; Sui et al., 2013; Varnum et al., 2014). Based on this research, it was predicted that the self-related processing effect can be augmented by independent self-construal. More specifically, the P2, N2, or P3 effects on self-related processing would be more prominent in independent than in interdependent self-construal groups.

## Materials and Methods

### Participants

A total of 224 undergraduate students were recruited to fill out the SCS (Singelis, 1994). This scale consists of two subscales that measure interdependent and independent self-construals on a 7-point Likert scale (1 = strongly disagree, 7 = strongly agree). It has been suggested that scores of self-construal can be computed by subtracting the mean scores of interdependence items from the mean scores of independence items, and participants with positive scores could be categorized as the independent self-construal group, and those with negative scores could be categorized as the interdependent self-construal group (Chiao et al., 2009; Liddell et al., 2015). Thus, according to previous studies, we subtracted the mean scores of interdependence items from those of independence items for each participant. Subsequently, participants who scored high (independent self-construal group; highest 15% of the distribution) and low (interdependent self-construal group; lowest 15% of the distribution) were chosen for further consideration. Eighteen subjects with independent self-construal and 20 subjects with interdependent self-construal were invited to attend the electrophysiological study (see Table 1). Data from one participant with independent self-construal was discarded due to excessive artifacts during electroencephalographic (EEG) recording. All participants were right-handed with normal or corrected-to-normal vision. This experiment was approved by local research ethics committees. Informed consent forms were obtained before the study, and payment was given after the experiment.

**TABLE 1:** Group demographic and self-construal scores.

	Group M(SD)	
	
	Independent self-construal	Interdependent self-construal
Age	21.15(1.53)	20.83(1.29)
Independence score	69.06(5.82)	50.2(7.08)
Interdependence score	57.06(6.58)	69.75(4.25)
Independence minus Interdependence score	12(6.05)	−19.55(5.84)

### Stimuli

The stimulus set comprised a small circle, a big circle, and names. The small circle and big circle were used as the target and standard stimulus, respectively. Participants’ own names and famous people’s names were presented visually and used as distracter stimuli. Specifically, we used two Chinese famous movie stars’ names (Jackie Chan and Andy Lau), and participants were familiar with these two persons. One famous-person name was used per subject. When the subject’s name was a two-character Chinese word, the Chinese name of Jackie Chan (a two-character name) was used as the famous name stimulus. When the subject’s name was a three-character Chinese word, the Chinese name of Andy Laud (a three-character name) was used as the famous name stimulus. Thus, the length was matched between the subject’s own and the famous person’s name.

### Experimental Task and Procedure

**FIGURE 1:**
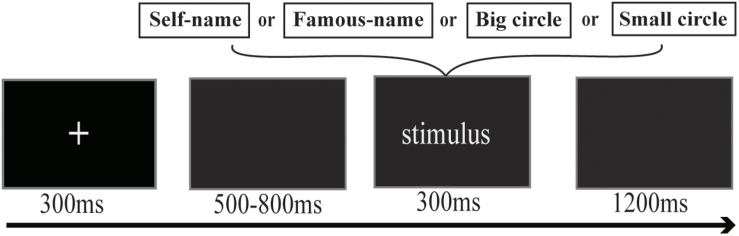
Procedure of the three-stimulus oddball task.

Subjects were seated in a soundproof ERP laboratory at a distance of 120 cm from the computer screen. Twenty-four practice trials were conducted before the formal experiment, which consisted of ten blocks. Each trial began with a fixation cross (300-ms duration) centered on the screen, followed by a gray screen presented for between 500 and 800 ms, randomly. Then, one of the four types of experimental stimuli, namely a big circle, a small circle, or one of the name stimuli, was presented for 300 ms. Subsequently, a gray screen was presented for 1200 ms (Figure 1). The big circle was presented 500 times, and each name stimulus and the small circle were presented 60 times each. Subjects were asked to identify and react as quickly and accurately as possible to the small circle if it appeared, with no need to respond to other stimuli. The name stimuli were equally distributed in ten blocks, with their sequential order being random in each block.

### Electrophysiological Data Recording and Analysis

The EEG data were recorded from 64 scalp electrodes mounted on an elastic cap (Brain Products) according to the extended International 10–20 system. The online reference is the FCz, and each electrode impedance was controlled to less than 10 kΩ. The offline EEG data were re-referenced to the mean of the bilateral mastoid electrodes and filtered with a 0.1–30 Hz Butterworth filter. The EEG data were epoched from 200 ms before stimulus presentation to 800 ms after its onset. Trials with amplitudes exceeding a threshold of ±80 μV were eliminated from the final averages.

Previous research has indicated that the P2, N2, and P3 components are sensitive to self-name processing (Zhao et al., 2009; Chen et al., 2011, 2015; Fan et al., 2013; Tacikowski et al., 2014). In addition, based on observation of the grand averaged waveforms, the P2 and N2 components are distributed mainly over the frontal and central areas, and the P3 component has an extensive scalp distribution, from the frontal to parietal regions of the brain. Thus, we analyzed three components, the P2 (160–220 ms) and N2 (260–300 ms) components with the F3, Fz, F4, FC3, FCz, FC4, C3, Cz, and C4 electrodes, and the P3 (350–550 ms) component with F3, Fz, F4, FC3, FCz, FC4, C3, Cz, C4, CP3, CPz, CP4, P3, Pz, and P4 as spatial regions of interest. Two-way repeated analyses of variance (ANOVA) were conducted on averaged amplitudes for these components, with self-construal group (independence vs. interdependence) as the between-subjects factor and the name type (self vs. famous) as the within-subjects factor. The ERP results were analyzed using ERPLAB toolbox (Javier and Luck, 2014). The *p*-values of all analyses were corrected by the Greenhouse-Geisser method.

## Results

**FIGURE 2:**
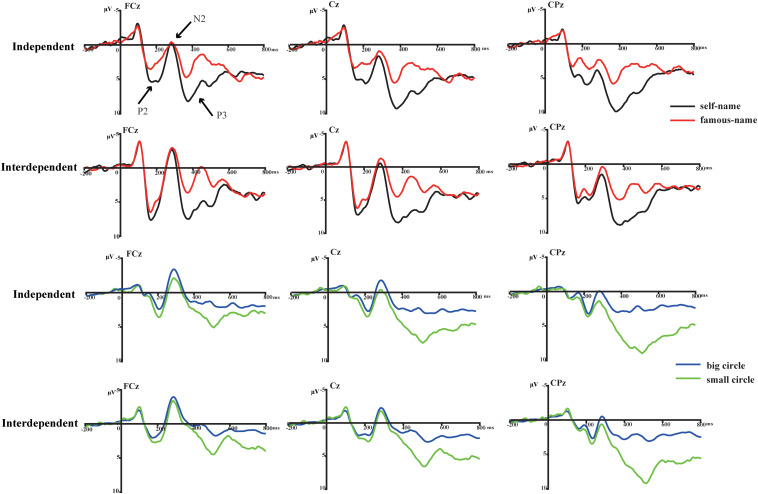
Grand averaged ERPs at FCz, Cz, and CPz to one’s own, a famous name, standard stimulus (a big circle), and target stimulus (a small circle) for groups with independent and interdependent self-construal.

**FIGURE 3:**
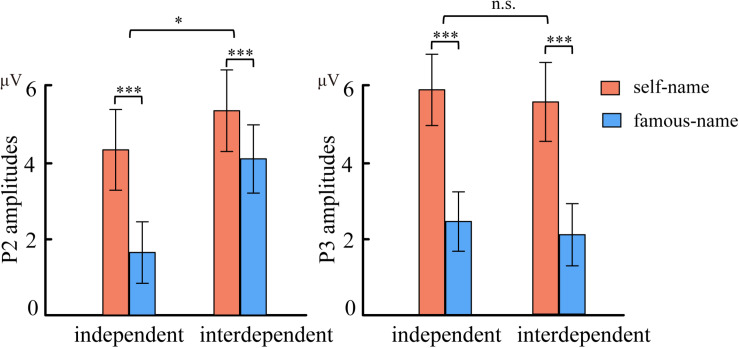
Mean amplitudes of the P2 (160–220 ms) and P3 (350–550 ms) components from self-name and famous name in independent and interdependent self-construal groups (***p < 0.001, *p < 0.05).

**FIGURE 4:**
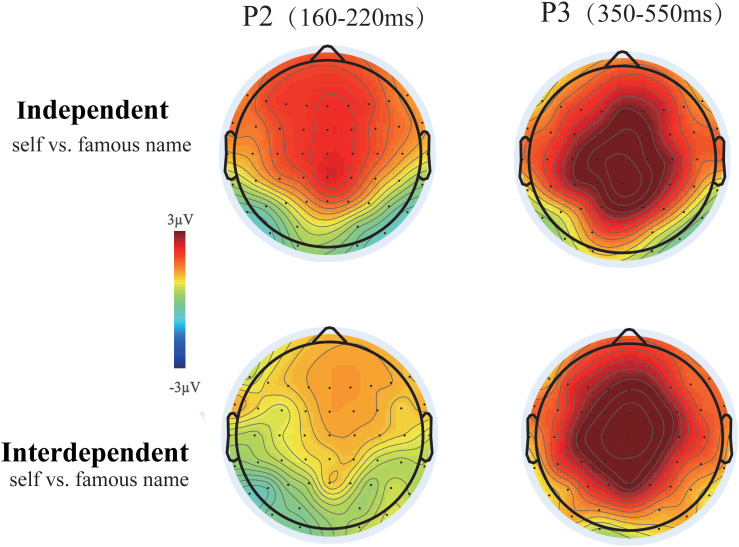
Topographic maps of own name minus famous person’s name ERPs at the P2 and P3 components for groups with independent and interdependent self-construal.

The ANOVA for P2 amplitudes indicated a significant main effect of name type [*F*(1,35) = 36.602, *P* < 0.001, $\eta_{p}^{2}$ = 0.511] at the frontal-central electrodes. Larger P2 amplitudes were observed for the subject’s own name (5.062 μV) than for the familiar name (3.404 μV) (see Figure 2). More importantly, the interaction effect of name type with self-construal group was also observed to be significant [*F*(1,35) = 4.765, *P* = 0.036, $\eta_{p}^{2}$ = 0.120] over the frontal-central area. Although the subject’s own name induced larger P2 amplitudes than the familiar name in both self-construal groups, the strength of self-processing bias, indexed by the P2 amplitude difference between the subject’s own and the familiar names, was larger in the independent than in the interdependent self-construal group (see Figure 3). Moreover, the modulation effects were mainly observed within the frontal-central area (see Figure 4).

In addition, both the main effect of name type [*F*(1,35) = 0.894, *P* = 0.351, $\eta_{p}^{2}$ = 0.025] and the name type by self-construal group interaction effect [*F*(1,35) = 0.029, *P* = 0.866, $\eta_{p}^{2}$ = 0.001] were not significant for the N2 amplitudes.

Although a significant main effect of name type was observed for P3 amplitudes [*F*(1,35) = 66.192, *P* < 0.001, $\eta_{p}^{2}$ = 0.654] over the frontal-central and parietal areas, the interaction effect between self-construal group and name type was not significant [*F*(1,35) = 0.001, *P* = 0.93, $\eta_{p}^{2}$ < 0.001] (see Figure 3).

## Discussion

This ERP study aimed to explore the influences of dispositional self-construal on neural responses to self-related processing. Our findings showed that self-name induced larger P2 and P3 amplitudes than did a famous person’s name in both independent and interdependent self-construal groups. No N2 amplitude differences were found between a subject’s own and the famous person’s name in either group. Moreover, the strength of the P2 effect (self vs. famous person’s name) was stronger in independent than in interdependent self-construal groups, and the strength of the P3 effect was similar between these two groups. The present study showed dispositional self-construal to had a modulation effect on self-related processing at the early P2 stage, indicating that an early self-processing advantage was much more prominent in individuals with independent self-construal.

Although self-name elicited larger P2 amplitudes compared with the famous person’s name in both groups, the P2 effect was more prominent in the independent self-construal group. The P2 component is related to the detection of emotionally significant stimuli, and increased P2 amplitudes reflect more attention resource allocation to these stimuli (Yuan et al., 2010; Carretié et al., 2011). In addition, the P2 component was found to be influenced by the extent of self-relevance and the importance of self-relevant content (Chen et al., 2011; Fan et al., 2013; Xu et al., 2017). For example, a previous ERP study showed that self-name (higher self-relevance) induced increased P2 amplitudes than lower self-relevance and non-self-relevant name stimuli (Fan et al., 2013). Moreover, one’s name is a symbol of identity, and its occurrence is related to potentially important events in everyday life (Watson, 1986; Tacikowski et al., 2013, 2014; Chen et al., 2015). Thus, one’s name is also a salient stimulus and can draw more attention than other names. In addition, it should be noted that the interaction between self-construal group and name type could also be broken down into the observations that smaller P2 amplitudes elicited by a famous name were observed in independent than in interdependent self-construal groups, whereas no significant differences in self-name condition between these two groups. It is possible that the priority processing of self-name might contribute to suppression of famous name processing, and thus a reduced P2 amplitude for famous name was observed in the independent self-construal group. These results indicated that individuals with independent self-construal demonstrated an enhanced self-related effect at the early P2 stage.

The N2 processing stage has been considered to reflect the frontier between automatic and controlled phases, and the N2 component has been related to the attention orienting response to emotionally salient stimuli (Carretié et al., 2004). We found no significant difference in N2 amplitude between self-name and famous name in either group, indicating that individuals with independent and interdependent self-construals had similar sensitivity to their names and famous names at the N2 processing stage.

We also observed that higher P3 amplitudes were evoked by self-name than by the famous person’s name in both groups, which was consistent with previous studies (Zhao et al., 2009; Fan et al., 2013) finding that self-name evoked higher P3 amplitudes compared to other name stimuli. Actually, the P3 component elicited by name stimuli was a novelty P3 component (P3a), which was an index of top-down controlled attentional process (Polich, 2007). Thus, larger P3 amplitudes for the participant’s own name demonstrated that enhanced amounts of top-down attentional resources were allocated to self-name. Moreover, the strength of the P3 effect was comparable between the independent and interdependent self-construal groups. Therefore, the modulation of dispositional self-construal on self-related effect might not occur at the higher-order cognitive stage.

Our findings have both similarities to and differences from previous studies regarding self-construal priming. For example, our findings were consistent with a previous fMRI study showing that priming independent self-construal could enhance activities in the right frontal regions for self-related processing (Sui and Han, 2007). In addition, Sui et al. (2013), using the self-construal priming paradigm, found that temporary priming of self-construal could modulate self-face processing at the N2 processing stage, whereas the present study provided evidence that influences of dispositional self-construal on self-name processing could occur as early as 160 ms after stimulus presentation, indexed by the P2 component. This seemed to reflect that dispositional self-construal demonstrated faster modulation on self-relevant processing than did temporarily activated self-construal. However, these differences might be due to the different self-relevant stimuli and experimental tasks adopted in these two studies. The self-name adopted in this study belongs to the psychological self, whereas the self-face adopted in Sui’s study belongs to the physical self (Liu et al., 2019). Furthermore, in addition to previous studies using face recognition or a self-reference task (Sui and Han, 2007; Chiao et al., 2009; Sui et al., 2013), our study provided further evidence on the modulation effect of self-construal on self-related processing in an oddball task.

## Conclusion

The present study demonstrated an obvious modulation effect of dispositional self-construal on neural responses in self-related processing at the P2 stage and found that the self-processing advantage was more prominent in individuals with independent self-construal. These findings might indicate fast modulation of self-relevant processing by dispositional self-construal.

## Data Availability Statement

The datasets generated for this study are available on request to the corresponding authors.

## Ethics Statement

The studies involving human participants were reviewed and approved by Ethics Committee of the Hunan Normal University. The patients/participants provided their written informed consent to participate in this study.

## Author Contributions

JC contributed the design of this study and collected the experimental data. CL, WL, and PY analyzed the data. JC, CL, and WL wrote the manuscript. CL, JC, YC, and PY revised the manuscript. All authors contributed and approved the final version of the manuscript.

## Conflict of Interest

The authors declare that the research was conducted in the absence of any commercial or financial relationships that could be construed as a potential conflict of interest.
